# *CePP2C19* confers tolerance to drought by regulating the ABA sensitivity in *Cyperus esculentus*

**DOI:** 10.1186/s12870-023-04522-2

**Published:** 2023-10-28

**Authors:** Jia Li, Xinyi Liu, Naveed Ahmad, Yifei Wang, Hengshuo Ge, Yijin Wang, Weican Liu, Xiaowei Li, Nan Wang, Fawei Wang, Yuanyuan Dong

**Affiliations:** 1 College of Life Sciences, Jilin Agricultural University, Changchun, 130118 China; 2 Joint Center for Single Cell Biology, School of Agriculture and Biology, Shanghai Jiao Tong University, Shanghai, 200240 China

**Keywords:** Protein phosphatase 2 C (PP2Cs), ABA signaling, Drought stress, Arabidopsis, Tiger nut

## Abstract

**Background:**

Tiger nut (*Cyperus esculentus*) is widely known as an additional source of food, oil and feed worldwide. The agricultural production of tiger nut has been greatly hindered by drought stress, reducing both yield and quality. Protein phosphatase 2 C (PP2Cs) plays an important role in plant responses to drought stress however, the molecular mechanism of PP2Cs in tiger nuts still unclear.

**Results:**

In this study, we identified a putative group A PP2C-encoding gene (*CePP2C19*) from tiger nut using transcriptome analysis, which is highly induced by drought stress. The transient expression assay suggested that *CePP2C19* was localized to nucleus. Furthermore, the interaction between CePP2C19 and CePYR1, a coreceptor for ABA signaling, was first detected using a yeast two-hybrid assay and then verified using a bimolecular fluorescence complementation (BiFC) analysis. In addition, the transgenic Arabidopsis lines overexpressing *CePP2C19* exhibited extreme tolerance to ABA and mannitol stresses during seed germination and root growth. At the mature stage, overexpression of *CePP2C19* resulted in a higher tolerance to drought stress in transgenic Arabidopsis, as confirmed by a visible phenotype and several physiological parameters. Noticeably, the silencing of *CePP2C19* by virus-induced gene silencing (VIGS) showed obvious reduction in drought tolerance in tiger nut plants.

**Conclusions:**

The *CePP2C19* emerges as a pivotal gene involved in the ABA signaling pathway, which likely reduce ABA sensitivity and thus enhances drought tolerance in *Cyperus esculentus*.

**Supplementary Information:**

The online version contains supplementary material available at 10.1186/s12870-023-04522-2.

## Introduction

Plants are often subjected to various biotic and abiotic stresses during their growth and development processes [1]. Drought conditions pose a severe threat to plant life as they impede growth and interfere with photosynthesis. This disruption subsequently retards the rate of carbon dioxide absorption, nutrient utilization, seed germination, and ultimately leads to yield loss [2, 3]. During extended periods of drought stress, plants exhibit a primary adaptive mechanism by accumulating osmotic compounds such as soluble sugars, proline, and glycine betaine. This accumulation serves the vital purpose of upholding cellular osmotic potential [4, 5]. A number of antioxidant enzymes, including malondialdehyde (MDA), superoxide dismutase (SOD), catalase (CAT), etc. have been demonstrated to undergo variations in their activity in response to drought stress. However, changes in plant physiological signals lead to variation in the expression of numerous drought-resistant genes [6, 7].

Protein kinases and phosphatases in plants play indispensable roles in the transmission of stress signals within plant cells. Based on the amino acid residues they dephosphorylate; this significant class of phosphatases is generally divided into serine/threonine (Ser/Thr) and tyrosine (Tyr) phosphatases. There are several subclasses of serine/threonine phosphatases, including PP1, PP2A, PP2B, and PP2C [8–10]. Prior studies have demonstrated that protein phosphatase 2Cs (PP2Cs) family is the major class of phosphatases with a plethora of biological processes related to ABA signaling cascade, environmental stresses, and plant defense [11, 12]. It is well-established that the phytohormone ABA plays a crucial role in the coordination of responses to abiotic stressors, particularly drought stress. During stress conditions, the process of ABA biosynthesis induces, and act as a dual negative regulatory mechanism. Group A PP2Cs, which consists of *ABI1, ABI2, Hab1, Hab2, AHG1*, and *PP2CA*, is assumed to be a suppressor of ABA signaling in *Arabidopsis thaliana* [13, 14]. Previous researches have shown that osmolarity-induced ABA alterations in plants may led to either conformational changes in ABA receptors such as pyrabactin resistance 1 (PYR1)/PYR1-like (PYL) or regulatory elements of ABA receptors such as (RCARs). As a result, PP2Cs dissociate from sucrose non-fermenting 1 (SNF1)-related protein kinase 2 (SnRK2s), allowing SnRK2s to autophosphorylate and activate downstream substrates such as bZIP transcription factor under stress condition [15–17]. Phosphorylation and dephosphorylation of target proteins are two essential mechanisms for efficiently activating and inhibiting key biochemical pathways. Positive modulators of ABA and osmotic response comprise subclass III SnRK2 kinases, which include *SnRK2.2, SnRK2.3*, and *SnRK2.6* [18–20]. Nevertheless, in the absence of ABA, PP2Cs inhibit the activity of SnRK2 proteins through physical interaction and dephosphorylation.

Prior to the discovery of a new ABA receptor (ABAR) in plants, PP2Cs were considered to be crucial players in ABA signal transduction [8]. Arabidopsis contains 14 members of ABARs, and 13 of which are involved in ABA sensing [21–23]. Several molecular interactions between Arabidopsis ABARs and PP2Cs have been discovered. For instance, members of group A PP2Cs such as (ABI1, HAB1, HAB2, and PP2CA) were de-activated in response to ABA, as shown by the functions of ABA receptors including PYR1, PYL1, PYL2, and PYR3 [24–26]. Interactions involving ABAR and PP2Cs have been found in several other higher plants than Arabidopsis, including rice, soybean, and maize. Rice ABA receptor OsPYL/RCAR5 was found to interact with group A PP2C, *OsPP2C30*, and a core ABA signaling which also comprised of a Ser/Thr kinase *SAPK2* [27–29]. In maize, the overexpression of *ZmPP2C55* resulted in improved drought stress as well as tolerance to salt, high temperature, and exogenous ABA responses [30]. In contrast, of the overexpression of OsPP108 demonstrated substantial improvements in physiological indices such as water loss, fresh weight, chlorophyll content, and photosynthetic potential (Fv/Fm) when subjected to salt or drought stress. These findings suggested that the overexpression of OsPP108 both positively influences abiotic stress responses and inversely modulates abscisic acid signaling [31].

Tiger nut is an herbaceous oil crop with rich oil content in its tubers and high nutritional value [32]. However, drought conditions greatly affect the yield and production of tiger nut, and therefore, it is of great practical importance to study the drought tolerance mechanisms in this plant. In this study, we identified a total 146 PP2C genes by transcriptome sequencing of tiger nut samples under different drought stress conditions. Based on the significant differential expression analysis, we identified a putative group A PP2C encoding gene (*CePP2C19*) from tiger nut. Further functional validation was conducted by cloning the full length of *CePP2C19* gene and then subcellular localization analysis, yeast two-hybrid and BiFC assay, overexpression in *Arabidopsis thaliana*, and gene silencing experiments, respectively. Altogether, our results demonstrated that the overexpression of CePP2C19-encoding gene leads to a high degree of drought tolerance in tiger nut by negatively regulating ABA signaling. The establishment of this platform will pave the wave in understanding the regulatory mechanism of a putative drought tolerant *CePP2C19* gene in tiger nut.

## Materials and methods

### Plant material and growth conditions

The plant material of tiger nut (*Cyperus esculentus* Hebei 01 cultivar, deposited in Engineering Research Center of Bioreactor and Pharmaceutical Development, Ministry of Education, JLAU) used in this study was identified by Jia Li. Seeds were soaked in water and placed in a constant temperature incubator at 37 °C to germinate. After 7 days, the uniformly growing seedlings were moved to 1/4 Hoagland nutrient solution for 10 days, and the hydroponic solution was changed every two days. Tobacco (*Nicotiana benthamiana*) seeds were planted and cultured in a soil mixture (peat moss, vermiculite, 1:1, v/v). Arabidopsis seeds (ecotype Col-0) were planted at 4 °C for 2 d after vernalization and cultured in a soil mixture (peat moss, vermiculite, 3:7, v/v). Overexpression of *CePP2C19* Arabidopsis seeds was grown in whole vermiculite after 2 d of vernalization at 4 °C. The *pp2c19* mutant is achieved by conducting the *AT2G29380* gene mutation in wild *Arabidopsis thaliana* (SALK-033011). The *pp2c19* mutant Arabidopsis seeds were grown in whole vermiculite after 2 d of vernalization at 4 °C under control conditions.

### Detection of physiological indicators of tiger nut and Arabidopsis

The leaves and roots were taken at 0 h, 12 h, 24 h, 48 h, and 72 h of stress treatment with 30% PEG6000 (Beijing Solarbio Science &Technology Co.,Ltd) and immediately placed in liquid nitrogen and stored in -80℃ refrigerator. The SOD, MDA, CAT, and soluble sugar contents were determined using physiological index test kits (Beijing Boxbio Science Technology Co., Ltd). The determination of ABA in tiger nut was performed using Shanghai Youxuan ABA ELISA kit. Fresh weight (Fw) of wild-type, mutant, and overexpression lines of *CePP2C19* in the control group and drought treatment group was measured and recorded. The Arabidopsis plants were de-greenized at 105℃ for 15 min and dried at 80℃ to constant weight (about 3 days) in the oven. Then, the dry weight (Dw) was weighed and recorded. Similarly, the water content of wild type and overexpression of *CePP2C19* Arabidopsis lines were calculated using the formula of water content (Fw - Dw)/ Dw *100%. Each group was tested for three biological replicates. In order to study the stomatal changes of Arabidopsis leaves after drought stress, Arabidopsis leaves were decolorized and fixed in ethanol: acetic acid (6:1) solution for 24 h, 30 min was treated with 70% ethanol dehydration, and anhydrous ethanol was treated 3 times, each time 30 min. Then the leaves were removed and transferred to the mixed solution of water and chloral [water and trichloro acetal: water: glycerol(w/v1/v2) = 8:1:2] for 8 h, and the stomata were observed by laser confocal microscope (Leica TCS SP8, Ger-many). Measurement of stomatal area was carried by ImageJ software.

### Differential expression analysis

Total RNA was obtained using the Fast-Pure Plant Total RNA Isolation Kit (Vazyme). Then, the paired-end sequencing using an Illumina NovaSeq 6000 platform (Illumina, San Diego, CA, USA) was conducted using three independent biological replicates for two samples. Following this, an assessment of the quality of the raw reads was performed using FastQC with the default settings. Additionally, the removal of adapter sequences was carried out using CutAdapt in order to obtain reads of high quality. The Trinity program was employed for the assembly of the transcriptome of tiger nuts. The contigs that were assembled were subjected to clustering based on the common reads of transcripts using Corset. From each cluster, representative sequences were identified and designated as unigenes. Furthermore, the functional classification within the Gene Ontology (GO) was conducted using WeGo. Additionally, for comprehensive functional annotation of unigenes, distinct sequences were mapped against multiple protein and nucleotide databases, including Nr, Nt, Pfam, COG, and Swiss-Prot. The calculation of transcript expression levels utilized the FPKM (Fragments Per Kilobase of transcript sequence per Millions of base pairs sequenced) method [33]. To control for false positives in multiple testing, the False Discovery Rate (FDR) correction was applied using DEseq2 [34]. Differentially expressed genes (DEGs) were identified based on criteria of |log2 (Fold Change) | > 1 and FDR < 0.05.

### Phylogenetic analysis of the PP2C gene family in tiger nut

Using tiger nut transcriptome data as well as rice and Arabidopsis public data, a phylogenetic tree was constructed by N-J method using MEGAX software with Bootstrap parameter set to 1000. The PP2C gene family of tiger nut was classified according to the Arabidopsis PP2C family gene classification pattern as well as rice. Tree files were visualized and modified using the online tools available at (https://itol.embl.de/).

### RNA isolation and qRT-PCR

Total RNA extraction was performed according to our previous method [35]. Then, the first-strand cDNA synthesis was carried out using the MonScript™ RTIII All-in-One Mix included in the dsDNase (Monad) kit. Gene primers for the tiger nut and Arabidopsis genes were generated with Premier 6 software (Premier Biosoft, Palo Alto, CA, USA) using the genomic sequence of each gene. To ensure the specificity of the primers, each primer pair was designed to target regions outside of the conserved domains of the genes. Subsequently, qRT-PCR was conducted using the MonAmp™ ChemoHS qPCR Mix from Monad Biotechnology Co. Ltd. (China) on a Stratagene MX3000P real-time PCR machine. In accordance with the manufacturer’s guidelines, a 20 µL reaction mixture was prepared, comprising 2.0 µL of cDNA template, 0.2 µM of primers (F/R), 0.2 µL of ROX dye (100x), 10 µL of master mix, and 7 µL of RNase-free water. Relative transcript abundance values were computed using the 2^−ΔΔCt^ method, with the tiger nut ADF7 (*CeADF7*) and Arabidopsis Actin3 (*AtACT3*) genes serving as standard controls for normalization.

### Drought and ABA treatments

Tiger nut were treated with 30% PEG6000 stress treatment. To examine *CePP2C19* expression in tiger nut. In the Arabidopsis plate experiment, 1/2 MS medium was added with 300 mM mannitol and 5 µM ABA.

### Yeast two-hybrid assay

The coding regions of *CePP2C19* and *CePYR1* were separately inserted into the pGBKT7 (BD) or pGADT7 (AD) vector. These construct pairs were then co-transfected into Y2HGOLD cells of the yeast strain. To evaluate the reliability of the protein interactions, we cultured yeast cells transformed with various concentrations (10^0^, 10^−1^, 10^−2^, and 10^−3^ dilutions) on SD medium deficient in Leu, Trp, His, and Ade (SD/−LTHA) supplemented with 5-bromo-4-chloro-3-indolyl-alpha-D-galactopyranoside (X-α-gal). After an incubation period of 3–4 days, we captured photographs of the resulting yeast growth. STRING website https://cn.string-db.org/.

### Bimolecular fluorescence complementation (BiFC) assay

The pxy104-CePYR1 recombinant vector and pxy106-CePP2C19 recombinant vector were constructed, and the recombinant plasmids were transferred into GV3101 (pSoup-p19) Agrobacterium receptor state, infiltrated with three-week-old leaves of *Nicotiana benthamiana*, cultured in dark for 24 h and then placed in an artificial climate chamber for normal growth, and GFP fluorescence was observed using confocal microscopy after 48 h.

### Subcellular localization

We conducted subcellular localization analysis by transiently expressing CePP2C19-GFP proteins in the leaves of *Nicotiana benthamiana*. The recombinant plasmids  constructed using *FlyCut* (TransGen Biotech) including pGDG-CePP2C19-GFP, pGDG-CePYR1-GFP, pGDG-GFP, and mCherry-NLS were introduced into Agrobacterium tumefaciens GV3101(pSoup-19) competent cells using the freeze-thaw method, individually. The leaves of *Nicotiana benthamiana* were subjected to transient expression through the injection of Trans-formed GV3101(pSoup-19). Following a 48 h incubation period under long-day conditions consisting of 16 h of light exposure, the cells were subjected to observation using a laser confocal microscope (Leica TCS SP8, Germany).

### Stress tolerance experiment of transgenic yeast

The INVSc1 yeast strain (Invitrogen, USA) was transformed with the expression vector and an empty pYES2 control plasmid using the lithium acetate technique [36]. The transformed cells were subsequently chosen for growth on uracil-deficient synthetic complete (SC) media supplemented with 2% (w/v) galactose at a temperature of 30 °C for a duration of 36 h. The assessment of osmotic tolerance was conducted in accordance with the previously published methodologies [37].

### Acquisition of overexpressing *Arabidopsis thaliana*

The pCAMBIA3301-CePP2C19 recombinant plasmid was constructed, and the recombinant plasmid was transferred into the EHA105 receptor state and infested with wild-type Arabidopsis thaliana in Colombia using the inflorescence infestation method (Floral-Dip) [38]. Screening pure strains for further research.

### Germination rate and soil phenotype analysis of overexpressed* Arabidopsis thaliana*

Arabidopsis seeds of the overexpression strain, mutant, and wild-type seeds were vernalized in a refrigerator at 4 °C, planted on 1/2 MS medium with ABA and mannitol stress, and placed in an incubator (16 h light, 8 h dark incubation) to observe changes in germination rate and root length within 6 days. The vernalized seeds, planted in whole vermiculite, were watered with 1/2 Hoagland solution when Arabidopsis grew to four rosette leaves, and drought stress was applied after three weeks.

### Virus-induced gene silencing assay

We employed the tobacco rattle virus (TRV)-based virus-induced gene silencing (VIGS) system to generate plants with silenced CePP2C19. Specifically, we transformed Agrobacterium rhizogenic strain GV3101 with pTRV1, pTRV2-CePP2C19, and pTRV2:00 (used as a negative control), and subsequently, these Agrobacterium cultures were introduced into tiger nut leaves.

### Statistical analysis

All experiments mentioned were conducted in triplicate, and the resulting data were subjected to statistical analysis using the t-test. A significant difference was defined as *P < 0.05 and **P < 0.01 and *** *P* < 0.001.

## Results

### Changes in the phenotypic and physiological indicators under drought stress in tiger nut

**Fig. 1:**
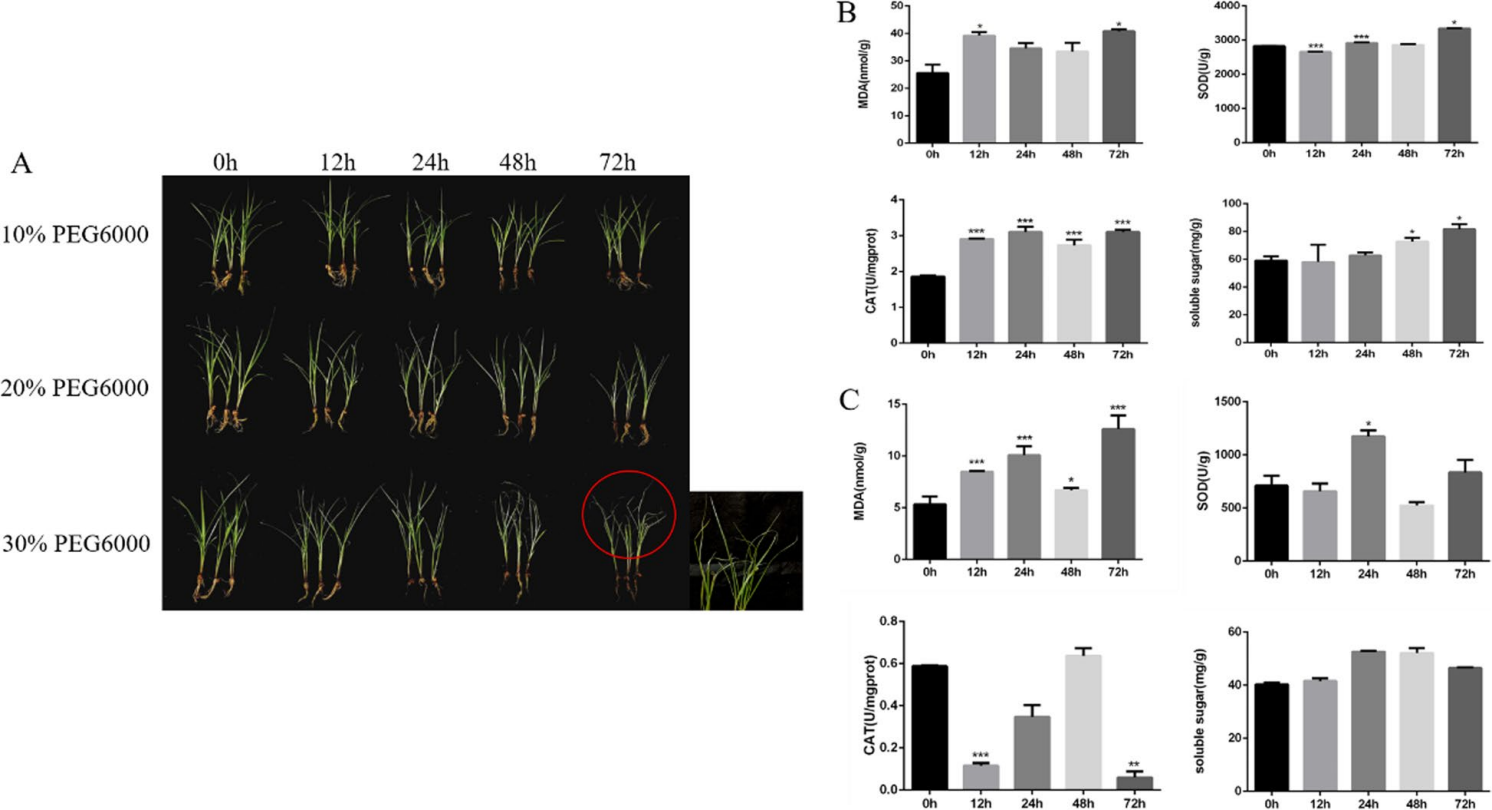
Determination of phenotypic and physiological indicators of tiger nut under drought stress. **A** The phenotype of tiger nut under different drought stress conditions at different time intervals (**B**) The contents of MDA, SOD, CAT, and soluble sugar in the leaves of tiger nut under 30% PEG6000 stress. Error bars represent the standard deviation. * Significant difference at *P* < 0.05 *** significant difference at *P* < 0.001. **C** The contents of MDA, SOD, CAT and soluble sugar in the roots of tiger nut under 30% PEG6000 stress. Error bars represent the standard deviation. * Significant difference at *P* < 0.05 **significant difference at *P* < 0.01 *** significant difference at *P* < 0.001

To investigate the effect of drought stress on tiger nut, simulated drought conditions were introduced using different concentrations of PEG6000 (10%, 20%, and 30%) at different time intervals (0 h, 12 h, 24 h, 48 h, and 72 h). Under 30% PEG6000 stress for 12 h, the leaves of tiger nut exhibited noticeable phenotypic changes including folding, wilting and water deficiency. Consequently, we assessed the physiological indicators of both the leaves and roots of tiger nuts under 30% PEG6000 stress (Fig. 1A). MDA content is an important index reflecting the degree of damage to the membrane system and the stress resistance of plants. The MDA content in leaves increased significantly (P < 0.05) after 30% PEG6000 treatment for 12 h. Moreover, the SOD activity also of different treatment times showed a significant difference. The activity of SOD was significantly reduced after 12 h of treatment but increased after 24 h of treatment. Similarly, the activity of CAT in the leaves was significantly enhanced at all time periods compared to the control treatment (*P* < 0.001). Furthermore, the content of soluble sugars in the leaves was significantly increased than the control group (*P* < 0.05) after 48 and 72 h of treatment (Fig. 1B). In the same way, these physiological indicators were investigated in the roots of tiger nut under 30% PEG6000 stress. The MDA content in roots was increased significantly (*P* < 0.05) compared to the control plants at all time points (Fig. 1C). SOD activity in the root tissues was significantly increased at 24 h treatment times. Moreover, the CAT activity in the roots was significantly decreased at 12 h (*P* < 0.001) and 72 h (*P* < 0.01) treatment times. The soluble sugar content in roots was slightly increased at all treatment times than the control group (Fig. 1C). Overall, the tiger nut leaves subject to 30% PEG6000 stress for 12 h treatment time was used for further analysis.

### Transcriptome data analysis

**Fig. 2:**
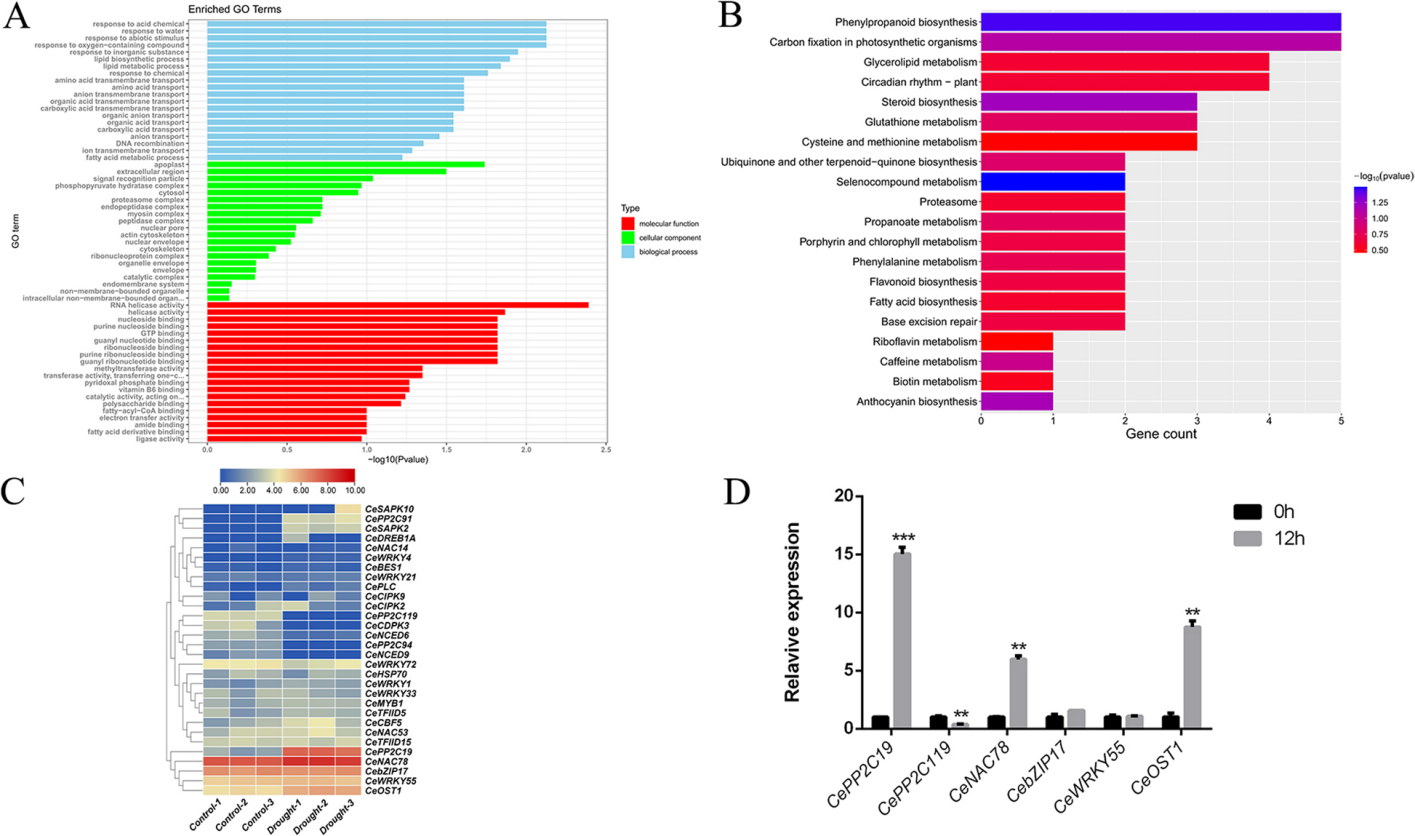
Transcriptome data analysis and functional annotation of DEGs identified in drought-induced stress of tiger nut. **A** GO enrichment analysis of the total DEGs were classified into three main categories: MF, CC, and BP. The names of the GO categories are listed along the y-axis. The degree of GO enrichment is represented by the -log_10_(*P*-value) and the number of transcripts enriched in each category. **B** Scatterplot of enriched KEGG pathways (www.kegg.jp/kegg/kegg1.html) of DEGs identified from drought stressed tiger nut leaves. Only the top 20 most strongly represented pathways are displayed. The color of the bar represents the range of the -log_10_(*P*-value). **C** Expression pattern of drought related genes. The colors indicate the abundance of transcripts calculated as Log_2_ (FPKM) in the control and drought-stressed plants (see color key). **D** Expression patterns of six genes in tiger nut leaves after treatment with 30% PEG6000 for 12 h. Error bars represent the standard deviation. ** significant difference at *P* < 0.01 *** significant difference at *P* < 0.001

To investigate the molecular mechanisms of plant interactions and coordination under drought stress, transcriptome sequencing of tiger nut leaves after 30% PEG6000 stress was performed. A total of 44,965 genes were annotated and 288 differentially expressed genes (DEGs) were identified with differential expression level in tiger nut. Of these DEGs, 196 were up-regulated and 92 were down-regulated in the leaves of tiger nut. We performed GO enrichment analysis of these DEGs, which classified them into three functional categories including biological process (BP), molecular function (MF), and cellular component (CC) (Fig. 2A). In the biological process category, the most significant enriched terms include response to water, response to abiotic stimulus, and response to oxygen-containing compounds. Similarly, the molecular function category showed the enrichment of the terms such as RNA-helicase activity, methyltransferase activity, ribonucleoside binding and catalytic activity. In case of cellular component, the go terms of apoplast, extracellular region, signal recognition particles and cytosol were mostly enriched. In addition, the top 20 functional KEGG enriched pathways of the drought-induced leaves of tiger nut were screened. Most number of DEGs were significantly enriched into the functional pathways including phenylpropanoid biosynthesis, selenocompound metabolism, carbon fixation in photosynthetic organisms, steroid biosynthesis, circadian rhythm plant, and anthocyanin biosynthesis (Fig. 2B).

Furthermore, thirty genes most likely related to drought stress tolerance were screened from the transcriptome data, and their differential expression pattern was investigated (Fig. 2C). Most of these genes such as *CePP2C94, CePP2C119, CeNAC14, CeWRKY21, CeWRKY33, CeMYB1, CeHSP70*, and *CeCIPK9* were down-regulated under drought stress conditions. However, genes encoding *CePP2C19, CeNAC78*, *CebZIP17, CeWRKY55* and *CeOST1* showed up-regulation under drought conditions when compared with the normal treatment group. To validate the integrity and stability of transcriptome data, we further conducted qRT-PCR assay of six genes under control and drought stress treatment. The results showed that the expression level of *CebZIP17*, and *CeWRKY55* was not significantly induced under both control and stressed condition. Similarly, gene-encoding *CePP2C119* also showed down-regulation in both control and stressed plants. Noticeably, the expression level of *CePP2C19* gene was high and significantly up-regulated under drought stress (Fig. 2D). Altogether, the analysis of transcriptome data and functional annotations of DEGs in drought-induced leaves of tiger nut suggested that *CePP2C19* could be the candidate gene in alleviating drought stress. 

### Phylogenetic analysis of the CePP2C family

**Fig. 3:**
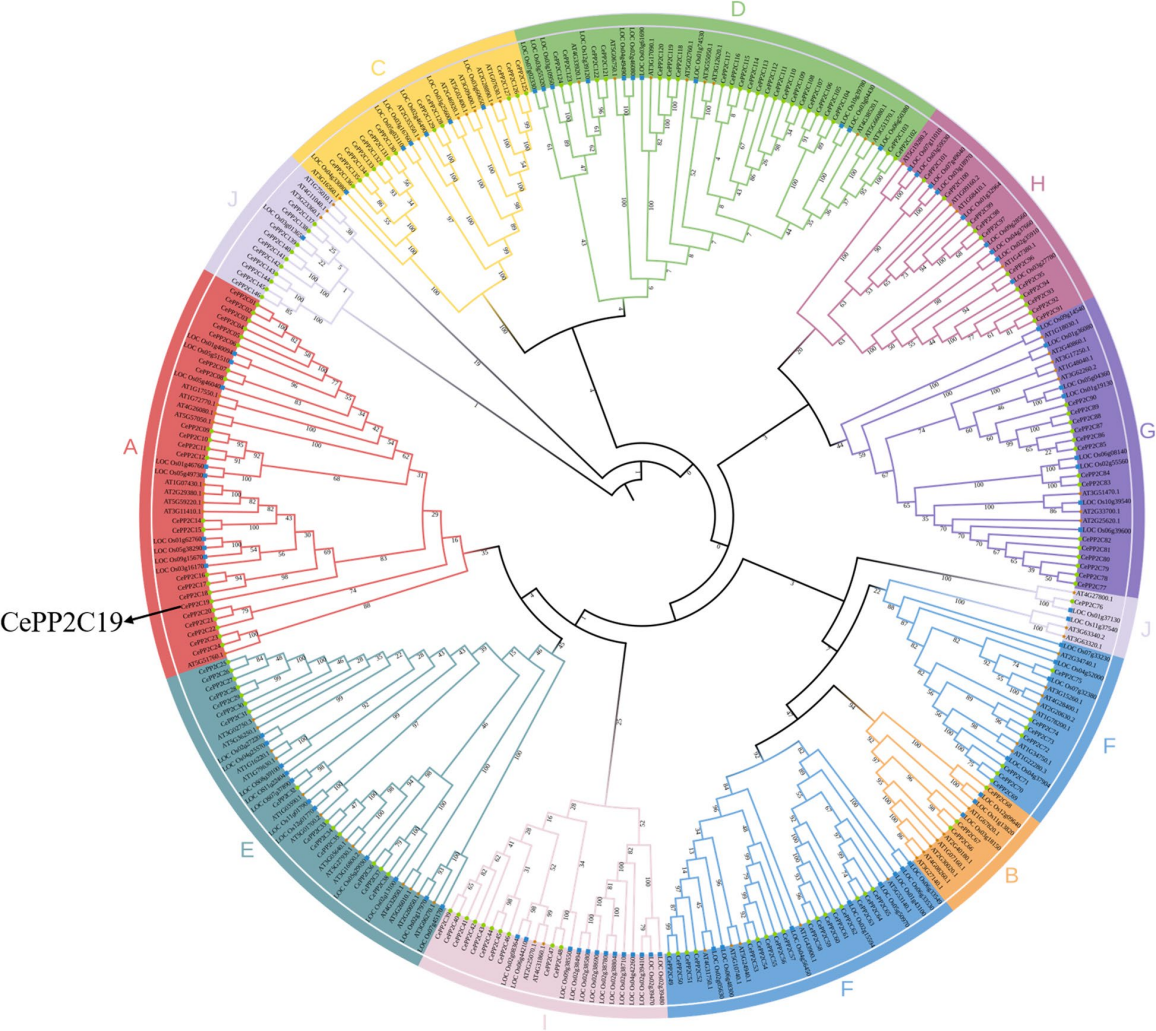
Phylogenetic analysis of PP2C members from *Arabidopsis thaliana*, *Oryza sativa*, and *Cyperus esculentus*. The phylogenetic tree was constructed by MEGA 6.0 with the neighbor-joining (NJ) method and 1000 bootstrap replications. Values less than 50 were removed. Members in the same clade with a unique color belong to the same subgroup

To explore evolutionary divergence and conserved relationships with other plant species, a phylogenetic tree of PP2C proteins was constructed among 146 *Cyperus esculentus* PP2C proteins, 76 *Arabidopsis thaliana* PP2C proteins, and 85 *Oryza sativa* PP2C proteins (Fig. 3). According to the classification pattern of the Arabidopsis family into A-J. Most of them belong to group A, group D and group F. Studies have shown that group A PP2C plays a decisive role in ABA signaling pathway. The phylogenetic analysis indicate that the tiger nut CePP2C19 belongs to the group A subfamily.

### CePP2C19 interacts with the key interactor partner CePYR1 in ABA signaling pathway

**Fig. 4:**
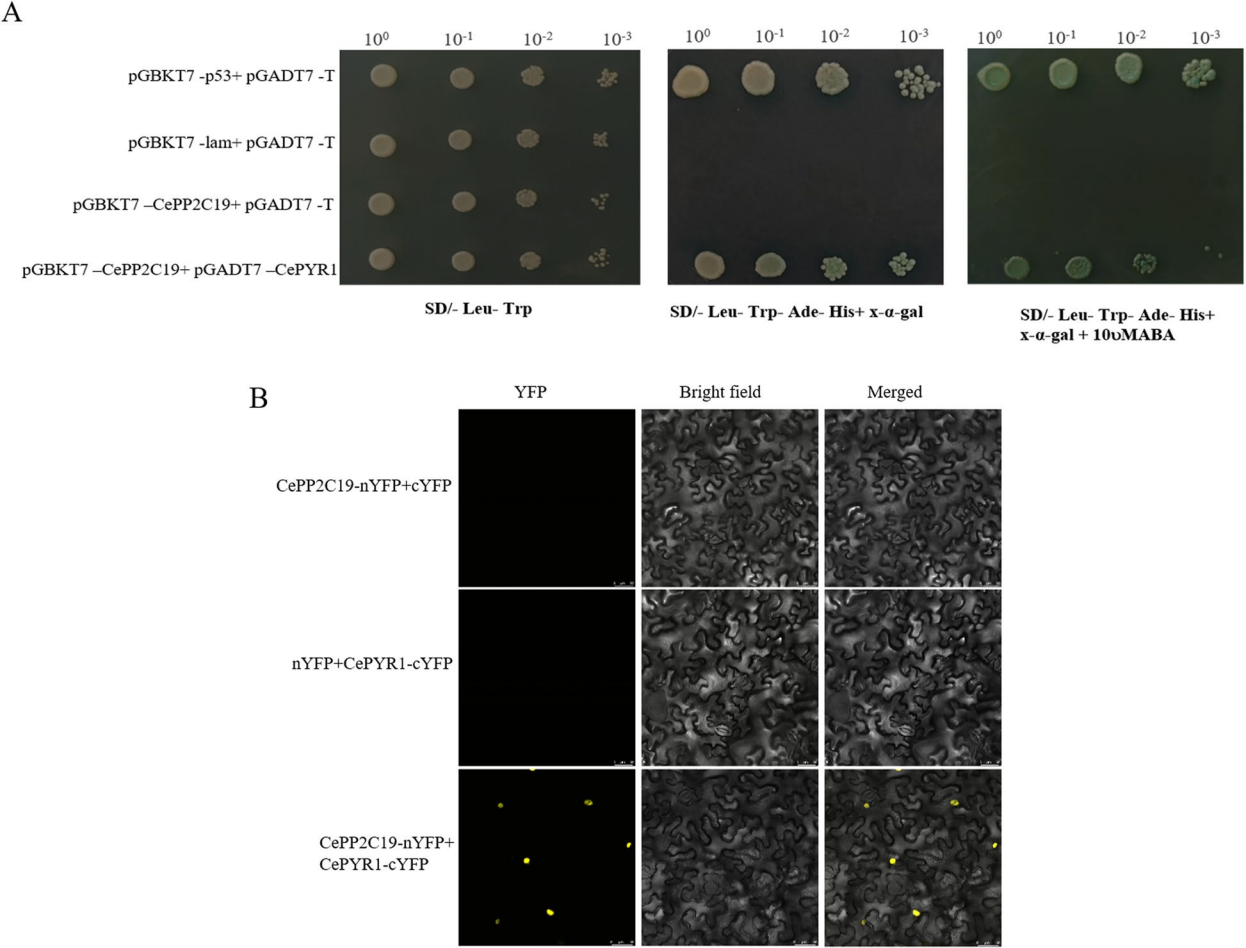
The interaction of CePP2C19 with CePYR1 receptor during ABA signaling. **A** Yeast-two-hybrid assay of CePP2C19 and CePYR1. **B** BiFC analysis. The fluorescence resulted from the complementation of the N-terminal portion of YFP fused to CePP2C19 (CePP2C19-nYFP) with the C-terminal portion of YFP fused to CePYR1. Fluorescence was observed in tobacco leaf epidermal cells. Scale bars = 50μm

To determine whether *CePP2C19* is a component of the ABA pathway, The primary predictions of protein interaction network was conducted using the online webserver of STRING. The results showed that *CePP2C19* is directly interacting with PYR1, a member of the widely known family of PYR/PYL/RCAR-ABA receptors. The expression of CePYR1 gene was up-regulated under drought stress (Figure S1). To determine whether the interaction of CePP2C19 with CePYR1 is dependent on exogenous ABA, the ABA solution was added to SD/-Leu-Trp-Ade-His medium. The initial evidence from yeast two-hybrid assay demonstrated the possible interaction between CePP2C19 and PYR1 that is unaffected by exogenous ABA (Fig. 4A). To further validate this interaction relationship, we performed a BiFC assay in tobacco leaves using YFP fusion (CePP2C19-YFP) construct. We produced DNA constructs in which CePP2C19 and CePYR1 were fused separately to the N-terminal (nYFP) and C-terminal (cYFP) parts of YFP containing vector. The results showed the appearance of yellow fluorescence in the nucleus, which further indicated that CePP2C19 and CePYR1 interact in the nucleus (Fig. 4B).

### Subcellular localization of CePP2C19 and CePYR1

**Fig. 5:**
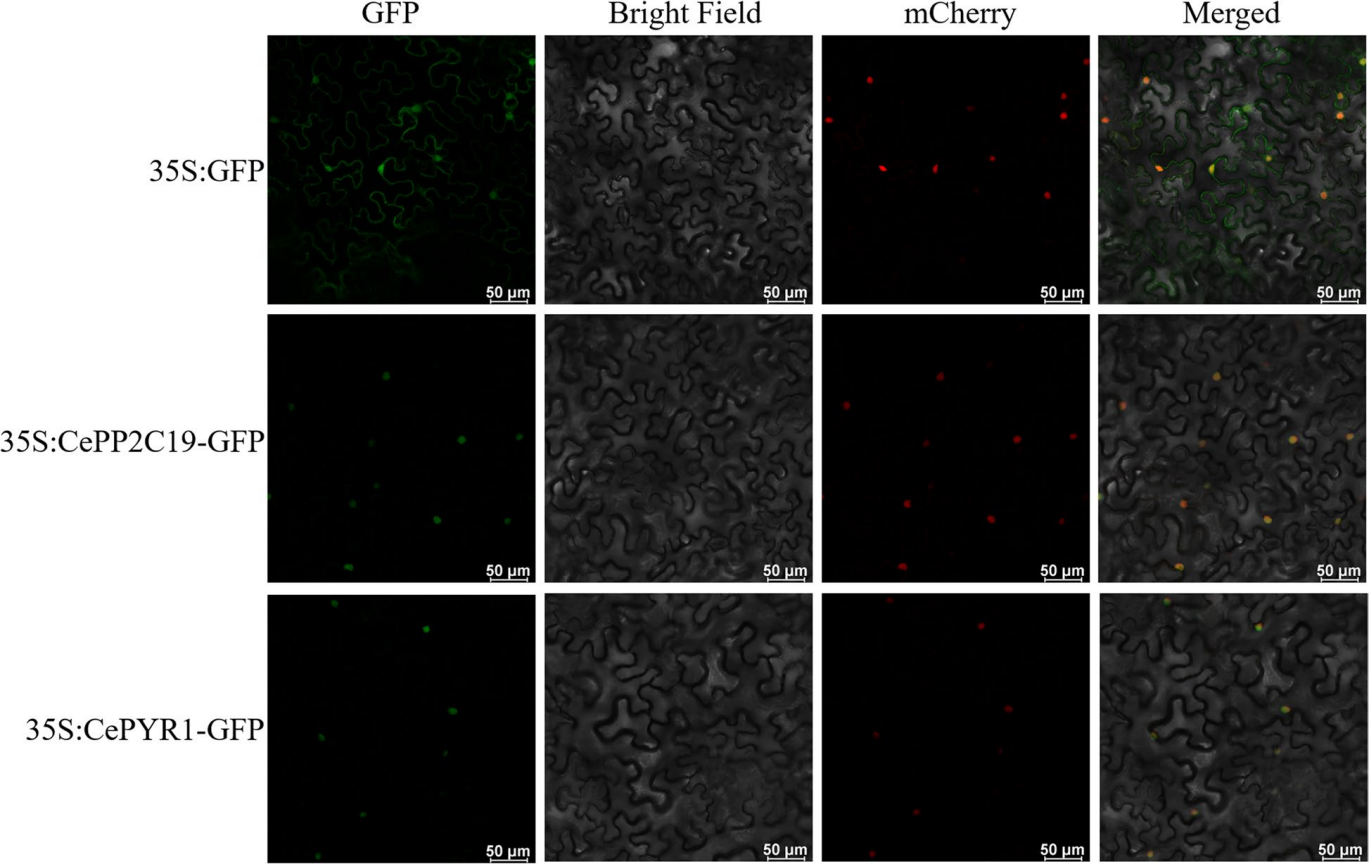
Subcellular localization of CePP2C19-GFP fusion proteins. Fusion proteins were transiently expressed under the control of the CaMV35S promoter in tobacco leaves and the results were observed under a laser scanning confocal microscope. The green color is the green fluorescent protein (GFP) signal. The red coloris a fluorescent protein mCherry-NLS. Scale Bars = 50 μm

The subcellular localization of CePP2C19 and CePYR1 were determined by utilizing the transient transformation system in tobacco leaves using the CePP2C19-GFP and CePYR1-GFP fusion construct. Confocal laser microscopy was used to examine the pattern of GFP fluorescence in the tobacco leaves. The subcellular localization of CePP2C19 and CePYR1 were examined through transient expression of CePP2C19-GFP and CePYR1-GFP construct in leaves of Nicotiana benthamiana plants with expressing a red nuclear location signal protein mCherry-NLS. The CePP2C19-GFP and CePYR1-GFP fusion were microscopically observed to be solely localized in nucleus, which was co-localized with a red fluorescent protein mCherry-NLS. In contrast, GFP alone was seen to be ubiquitously distributed throughout the cell without specific localization. These results indicate that CePP2C19 and CePYR1 are a nucleus-localized protein (Fig. 5).

### Functional verification of CePP2C19 in yeast

**Fig. 6:**
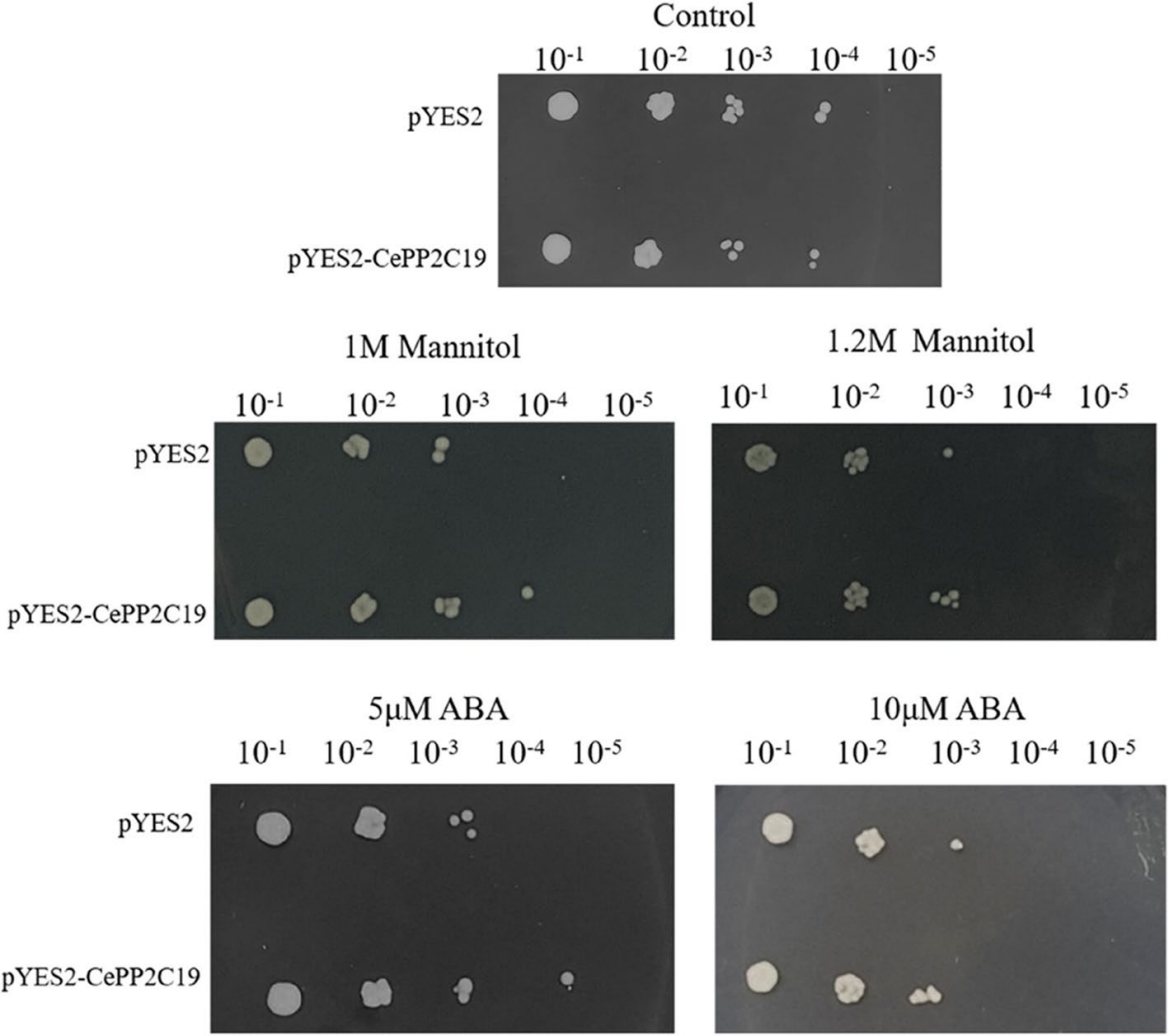
Phenotypic growth assays of *Saccharomyces cerevisiae* INVSc1 cells transformed with the pYES2 empty vector, pYES2-CePP2C19 under drought stress and ABA stress. Yeast cells transformed with the pYES2 empty vector, pYES2-CePP2C19, were spotted on SC-Ura medium 10-fold serially diluted (10^−1^, 10^−2^, 10^−3^,10^−4^, and 10^−5^) cultures and were then incubated at 30 °C for 36 h

The heterologously overexpressed *CePP2C19* gene in the yeast strain INVSc1 using the pYES2 vector was further investigated for its possible role under the exposure of mannitol and ABA stress. The results showed no significant difference in the survival rates between the pYES2 and *CePP2C19* transgenic yeast cells under non-stress conditions. However, after 36 h of incubation in 1 M mannitol and 1.2 M mannitol, the transformed lines survived better than the control lines. Similarly, the transformed lines also survived better than the control lines after 36 h of incubation in the medium supplemented with ABA stress (Fig. 6). The results suggest that CePP2C19 protein may confer drought tolerance and ABA tolerance to yeast cells.

### Overexpression of *CePP2C19 *confers abiotic stress tolerance in Arabidopsis seedlings

**Fig. 7:**
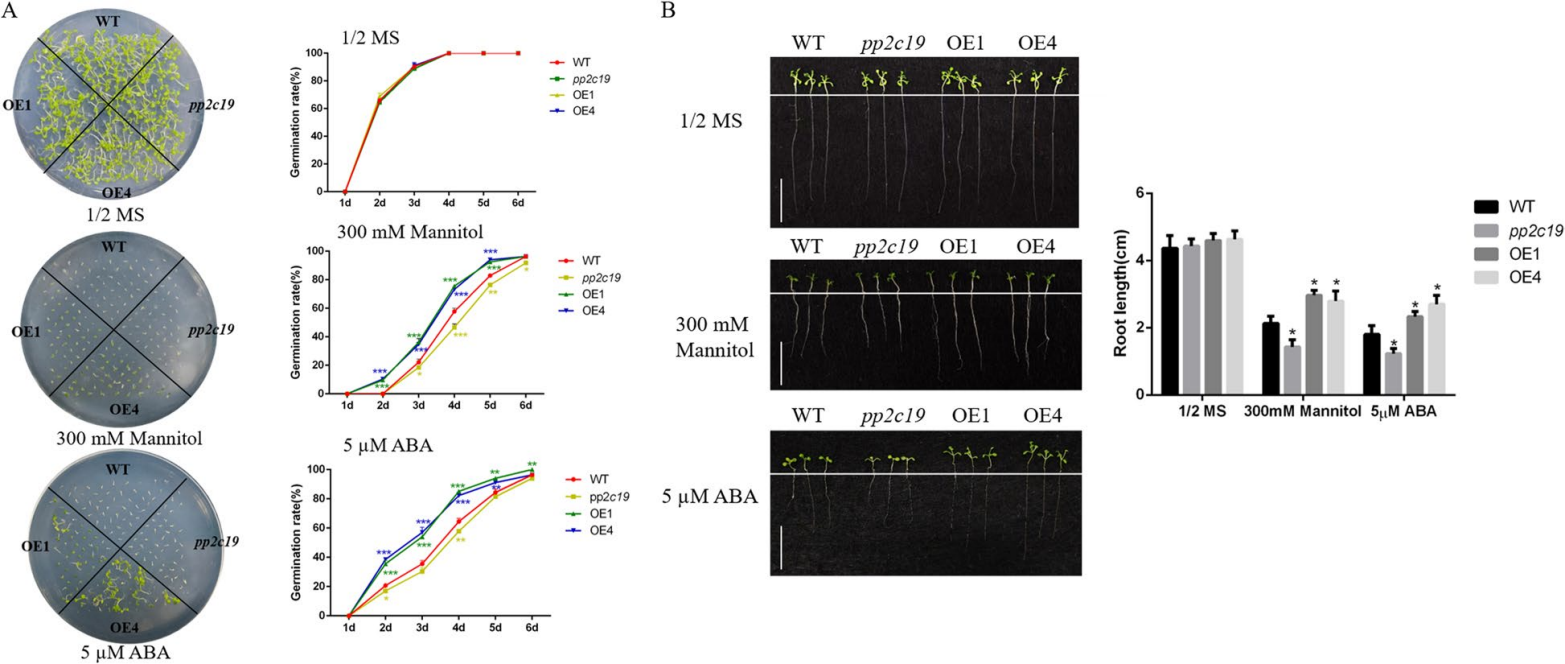
Overexpression of *CePP2C19* improved the tolerance of Arabidopsis seedlings to drought and ABA stress. **A** Seed germination under the treatment of mannitol and ABA. The surface-sterilized Arabidopsis seeds of wild-type and overexpression lines were sown on solid media of 1/2 MS containing mannitol (0, 300 mM) and ABA (5 µM). The status of seed germination was taken by photos after the treatment of stress for 6 days, respectively, while the germination rate of seeds was counted every day. WT (wild type); *pp2c19* (mutant); OE1, OE4 (overexpression lines). Error bars represent the standard deviation. * significant difference at *P* <0.05 ** significant difference at *P* < 0.01 *** significant difference at *P* < 0.001. **B** Seedlings under the treatment of mannitol and ABA. WT (wild type); *pp2C19* (mutant); OE1, OE4 (overexpression lines). Error bars represent the standard deviation. * significant difference at *P* <0.05

We performed seed germination and root growth assays of wild-type (WT), *pp2c19* mutant and two overexpression lines (OE1 and OE4) of *CePP2C19* on 1/2 MS medium supplemented with mannitol and ABA. Our statistical results showed that mannitol and ABA supply leads to an inhibitory effect on seed germination rate of transgenic lines. As described in Fig. 7A, no obvious difference was detected in the germination rates of WT, *pp2c19* mutant, and overexpression strains on 1/2 MS medium alone. However, the overexpression lines started to germinate on the second day, while the WT and *pp2c19* mutant lines did not germinate under mannitol stress. Noticeably, the germination rate of the overexpression lines was significantly faster than that of the WT. Importantly, the germination rate of the overexpression lines was faster than that of the WT and the mutant at 2–5 days under ABA stress, and the germination rate of the mutant was lower than that of the WT. Furthermore, the overexpression lines exhibited a significant increase in root length compared to the WT and mutant lines under mannitol stress conditions (Fig. 7B). In the same way, the root length of the overexpression lines was longer than that of the WT and mutant lines under ABA exposure. Collectively, these results suggested that the overexpression of *CePP2C19* enhance drought tolerance and decrease ABA sensitivity in Arabidopsis, which might play a kay role during the regulation of ABA signaling at the germination stages.

### Overexpression of *CePP2C19* confers drought tolerance in mature Arabidopsis plants

**Fig. 8:**
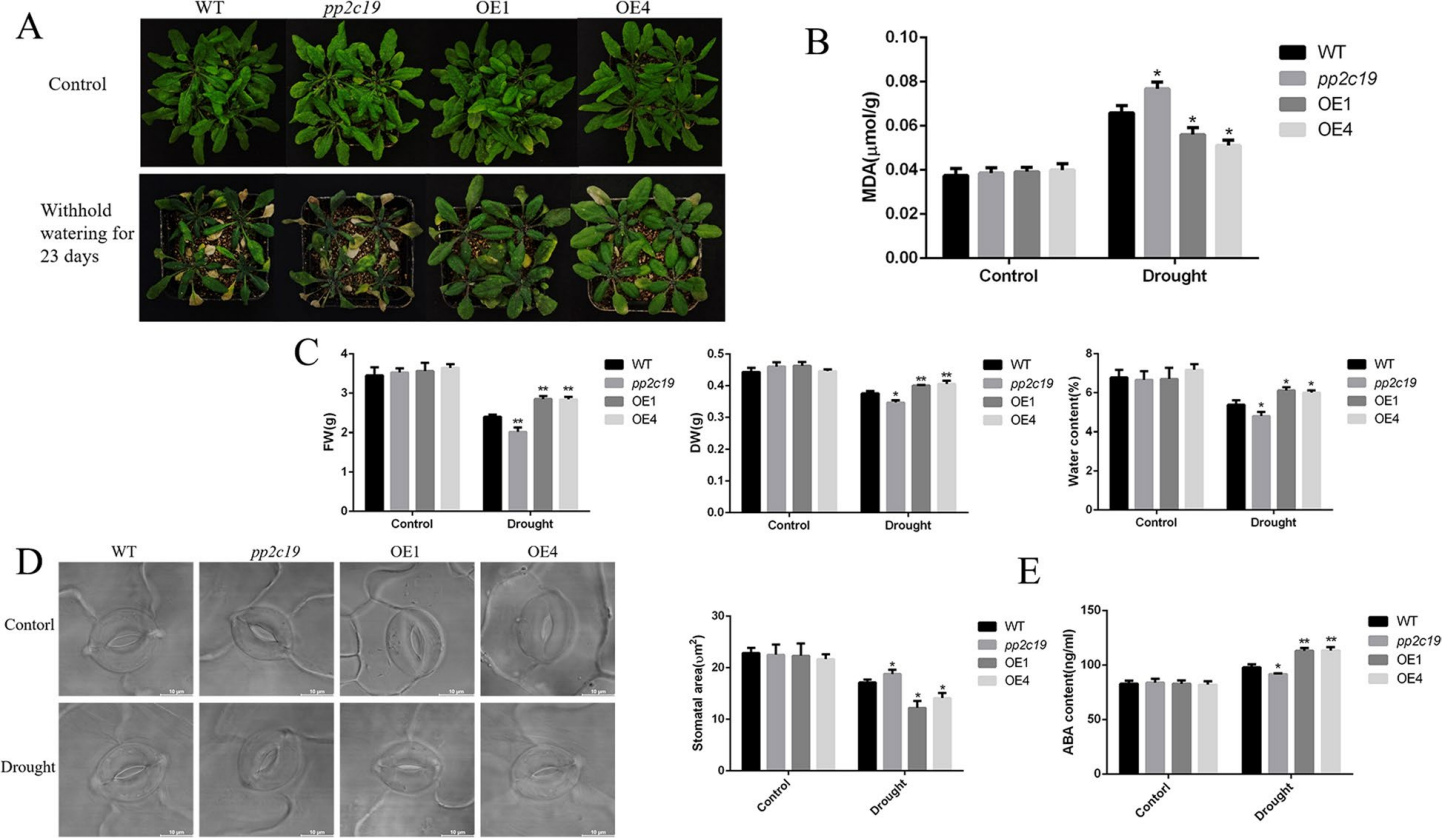
Overexpression of *CePP2C19* enhances drought tolerance in mature Arabidopsis plants. **A** The phenotypes of *CePP2C19* transgenic lines tolerant to drought stress. **B** The determination of MDA content in the WT (wild type); *pp2C19* (mutant); OE1, OE4 (overexpression lines), Error bars represent the standard deviation. * Significant difference at *P* < 0.05. **C** The determination of fresh weight, dry weight, and moisture content. Arabidopsis seeds of WT，mutant and *CePP2C19 *transgenic lines of the T3 generation were sown in vermiculite and cultured in the chamber room. WT (wild type); *pp2C19* (mutant); OE1, OE4 (overexpression lines). Error bars represent the standard deviation. The asterisk indicates * significant difference at *P* < 0.05 **significant difference at *P* < 0.01. **D** Stomatal image and stomatal area of Arabidopsis thaliana leaves under drought stress. WT (wild type); *pp2C19* (mutant); OE1, OE4 (overexpression lines). Scale Bars = 10 µm. Error bars represent the standard deviation. * significant difference at *P* < 0.05 **E** Determination of ABA content in Arabidopsis. WT (wild type); *pp2C19* (mutant); OE1, OE4 (overexpression lines) Error bars represent the standard deviation. *Significant difference at *P* < 0.05. **significant difference at *P* < 0.01

To investigate the effect of drought stress on mature transgenic Arabidopsis plants, we conducted phenotypic and physiological analysis of *CePP2C19* overexpression lines, mutant and WT lines. After a period of 23 days of drought treatment, it was observed that the leaves of both WT and mutant plants had become dry, yellowed, and wilted. However, the overexpression lines exhibited a healthier appearance with less yellowing of the leaves (Fig. 8A). In addition, the amount of MDA accumulation in transgenic Arabidopsis was found to be lower than that of WT Arabidopsis after drought stress (Fig. 8B). However, the fresh weight, dry weight, and water content of *CePP2C19* transgenic lines were more than that of WT plants (Fig. 8C). Moreover, under drought stress, the stomatal area of transgenic Arabidopsis was significantly smaller compared with WT (Fig. 8D). The ABA content of transgenic Arabidopsis lines was significantly higher than WT Arabidopsis under drought stress (Fig. 8E). These results showed that the transgenic Arabidopsis demonstrated less damage and showed considerable ability to resist drought stress.

### Reduced drought tolerance in CePP2C19-silenced tiger nut

**Fig. 9:**
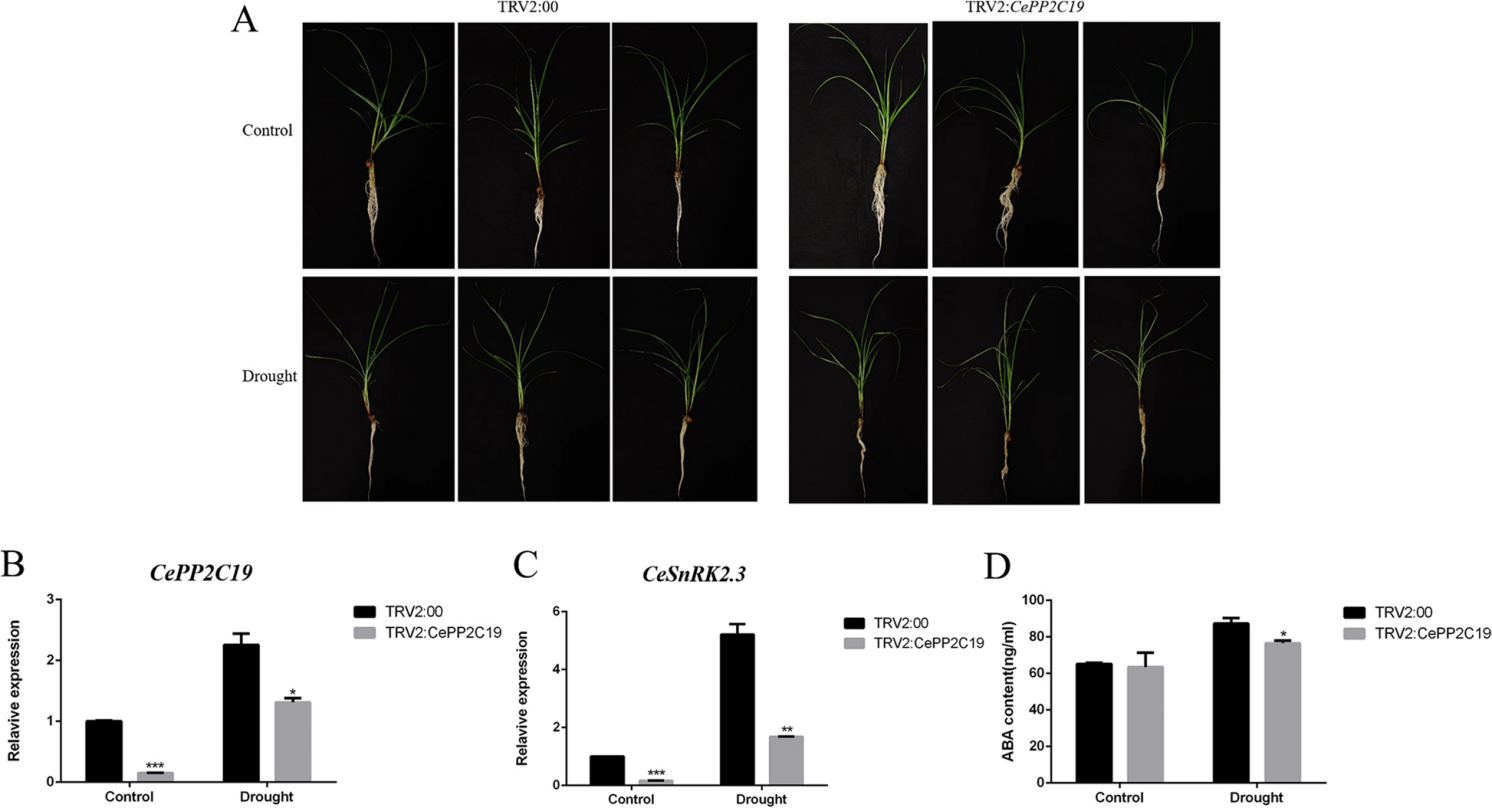
Reduced drought tolerance of TRV2:*CePP2C19* in tiger nut. **A** Drought stress with 30% PEG6000 on TRV2:*CePP2C19* and TRV2:00 plants. **B** qRT-PCR analysis of *CePP2C19* expression in leaves of TRV2:00 and TRV2:*CePP2C19* plants exposed to drought stress. Relative expression level of each gene was normalized to that of the tiger nut *ADF7* (*CeADF7*) as a standard control. Error bars represent the standard deviation. * Significant difference at *P* < 0.05 *** significant difference at *P* < 0.001. **C** qRT-PCR analysis of *CeSnRK2.3* expression in leaves of TRV2:00 and TRV2:*CePP2C19* plants exposed to drought stress. Relative expression level was normalized to that of the tiger nut *ADF7* (*CeADF7*) as a standard control. Error bars represent the standard deviation. **significant difference at *P* < 0.01 *** significant difference at *P* < 0.001 (D) Determination of ABA content in TRV2:00 and TRV2:*CePP2C19*. Error bars represent the standard deviation. *Significant difference at *P* < 0.05

As the stable transformation system of tiger nut has not yet been fully developed, we performed the phenotypic analysis of *CePP2C19-*silenced plants using virus-induced gene silencing (VIGS). We subjected TRV2:*CePP2C19* (silenced) containing plants and TRV2:00 (control) to 30% PEG6000 stress. Notably, the TRV2:*CePP2C19* exhibited a more pronounced withering phenotype compared to the TRV2:00 plants (Fig. 9A). Likewise, the expression level of *CePP2C19* was down-regulated under drought stress in the *CePP2C19*-silenced tiger nut (TRV2:*CePP2C19*) as opposed to the control tiger nut (TRV2:00) (Fig. 9B). Furthermore, the expression level of *SnRK2* gene was observed in TRV2:*CePP2C19* plants and TRV2:00 plants. The *SnRK2* gene, known as a central gene in drought, salt, and ABA signaling, served as a positive control. Prior to drought exposure, TRV2:*CePP2C19* plants displayed a noteworthy reduction in *CeSnRK2.3* expression in comparison to TRV2:00 plants. However, both plant lines exhibited an upregulation of *CeSnRK2.3* expression post drought treatment. Notably, the expression levels of *CeSnRK2.3* were considerably lower in TRV2:*CePP2C19* plants compared to TRV2:00 plants (Fig. 9C). Additionally, we quantified the ABA content in TRV2:*CePP2C19* and TRV2:00 plants after drought stress, the results indicated a lower ABA content in TRV2:*CePP2C19* plants compared to TRV2:00 plants following drought stress (Fig. 9D). Collectively, these results indicated that *CePP2C19* could be involved in the molecular regulatory mechanism of drought stress tolerance by exploiting ABA signaling pathway in tiger nut.

## Discussion

Drought is an important factor limiting the yield of *Cyperus esculentus* (tiger nut). Therefore, understanding the molecular and genetic basis of drought resistance in tiger nut is an urgent need for breeding drought tolerant varieties. By triggering the activation of a multitude of stress-related genes, ABA orchestrates the plant’s adaptive response to abiotic stress, particularly in conditions of water scarcity. When it comes to cell differentiation and stress signaling, PP2Cs are the crucial regulators among others [39]. Many PP2C genes have now been discovered throughout plant taxa. Several members of group A PP2Cs play important roles in regulating ABA signaling in Arabidopsis, especially when the plant is subjected to abiotic stressors. A parallel signaling cascade was emerged in rice and maize [40–42]. Admittedly, there is a lack of publicly available research on tiger nut and it is unclear what role it plays under different stress conditions. In this study, we provide the first report of in planta functional characterization of a group A PP2Cs from tiger nuts, which likely play important role in a signaling module involved in drought stress and ABA signaling.

We identified a total of 146 PP2C members from the transcriptome data of tiger nuts. Phylogenetic analysis showed that *CePP2C19* belongs to group A of tiger nut PP2C, and its sequence is highly homologous to members of the ABA-related Arabidopsis group A PP2C. Moreover, subcellular localization in *Nicotiana benthamiana* suggested that CePP2C19 was a nuclear protein. Consistent with the previous study, the rice PP2C subfamily group A member OsPP108 was likewise shown to be nuclear-localized [31]. Furthermore, the qRT-PCR analysis distinctly illustrates the induced expression of *CePP2C19* genes to drought stress. This additional substantiation significantly underscores the assertion that *CePP2C19* genes actively contribute to enhancing stress tolerance mechanisms. Other components such as the complex of ABA signaling components (PYR/PYL receptors, PP2Cs, and Ser/Thr Kinase) which is formed in the nucleus, also regulate ABA-mediated gene expression [43]. The interaction between PP2C and the ABA receptor PYL is the key step that triggers the downstream signaling genes to evoke ABA signaling [44, 45]. This signaling network has also been reported in plant species such as Arabidopsis, maize, tomato, and cotton. Many studies have shown that group A PP2C can interact with the ABA receptor family. For example, ZmPLY3 and ZmPP2C16 proteins in maize interact in response to ABA induction, demonstrating that they may be the most likely members of the second component and receptor of the ABA signaling pathway [46]. The HAI PP2Cs interacted with PYL5 and PYL7-10 in Arabidopsis [47]. AHG3 interacts with PYL12 in response to ABA and functions specifically in seed germination and early seedling growth [48]. The Cotton GhPYLs are effective against the phosphatase activity of GhHAI2, GhAHG3, and GhABI2 [49]. We also found that CePP2C19 interacts with CePYR1, a member of the ABA receptor family, and provided further evidence that CePP2C19 is pivotal to ABA signaling cascade.

We performed a preliminary validation of the *CePP2C19* gene for drought tolerance and ABA tolerance in a yeast system. Previously, the overexpression of four transcription factors from vetch plant in transgenic yeast enhanced yeast tolerance to osmotic stress [37]. In our further findings, the overexpression of *CePP2C19* gene in Arabidopsis showed drought tolerance and ABA sensitivity on MS medium. Under stress, the transgenic lines showed significant differences compared with WT, with the transgenic lines showing a faster germination rate and longer root length than WT. In addition, phenotypic experiments in soil experiments similarly demonstrated this difference. Under water deficit stress, complicated regulation processes are activated in plants, including at the biochemical, physiological, and molecular levels [50]. MDA content is an indicator of lipid peroxidation. MDA was measured and the MDA values of the transgenic strains were all less than WT, indicating that the membranes were damaged to a lesser extent and were drought tolerant. Fresh weight, dry seeds, and water content are also important indicators of drought tolerance of plants. The dry and fresh weights and water contents of the transgenic strains were higher than those of WT. The VIGS method has been widely used in many plants to interfere with genes to obtain mutant plants [51]. We used VIGS genetic analysis in tiger nut to further understand the function of *CePP2C19* gene. In our phenotypic experiments, plants with *CePP2C19*- silenced showed a sensitivity to drought, which was followed by a reduction in ABA levels. Taken together, these data led to the conclusion that low *CePP2C19* expression reduces the plant’s ability to withstand drought. Reducing the expression levels of GhHAI2, GhAHG3, and GhABI2 in cotton has been shown to increase the plant tolerance to osmotic stress [49]. Group A PP2Cs inhibit the ABA signaling pathway by dephosphorylating SnRK2 type kinases, which leads to the degradation of these proteins [52]. The conducted qRT–PCR analysis was undertaken with the specific aim of elucidating the causal relationship between variations in the *CePP2C19* gene and the ensuing changes in the downstream gene *SnRK2.3*. The results yielded from this investigation distinctly demonstrate that the presence of the *CePP2C19* gene does exert a discernible influence on the alterations observed in the downstream gene cascade. This observation substantiates the intricate interplay between these genes within the broader regulatory network. This pivotal role is testament to the intricate web of molecular interactions that orchestrate a plant’s response to drought stress, with CePP2C19 occupying a central position in this intricate regulatory framework.

## Conclusion

The comprehensive understanding of a nuclear-localized CePP2C19 unfolds crucial insights into the intricate defense mechanisms that tiger nuts deploy when confronted with drought stress. Notably, the augmentation of *CePP2C19* overexpression distinctly influences seed germination patterns and augments drought tolerance, especially during the early seedling stage, accompanied by a notable reduction in ABA sensitivity. This multifaceted response underscores the critical role of CePP2C19 as a key mediator of stress adaptation. Envisaging the broader agricultural context, the strategic overexpression of *CePP2C19* emerges as a promising avenue to mitigate the vulnerability of plants to adverse conditions in their natural habitat. This approach holds the potential to foster elevated crop yields and enhanced productivity, thereby contributing significantly to the sustainability and resilience of agricultural systems in challenging environments.

### Supplementary Information

**Additional file 1: Figure S1.** qRT-PCR analysis of *CePYR1 *expression in leaves of tiger nut exposed to drought stress. Error bars represent the standard deviation. **significant difference at *P* < 0.01

## Data Availability

The RNA sequencing data for both drought stress and non-drought stress tiger nut samples were deposited in the SRA database at the NCBI under accession number PRJNA975975. All other data and material analyzed in the current study are included in the manuscript and the supplementary information.

## Abbreviations

PP2C: Protein phosphatase 2C
MDA: Malondialdehyde
SOD: Superoxide Dismutase
CAT: CATalase
DEGs: Differentially expressed genes
BiFC: Bimolecular fluorescence complementation
VIGS: Virus-induced gene silencing
