# Supplementary material for: CePP2C19 confers tolerance to drought by regulating the ABA sensitivity in Cyperus esculentus
Source: BMC Plant Biol. 2023 Oct 28;23:524. doi: 10.1186/s12870-023-04522-2 (PMC10612301; doi:10.1186/s12870-023-04522-2)
Supplement: Supplementary file 1 — Additional file 1: Figure S1. qRT-PCR analysis of CePYR1 expression in leaves of tiger nut exposed to drought stress. Error bars represent the standard deviation. **significant difference at P < 0.01 [file 12870_2023_4522_MOESM1_ESM.docx]

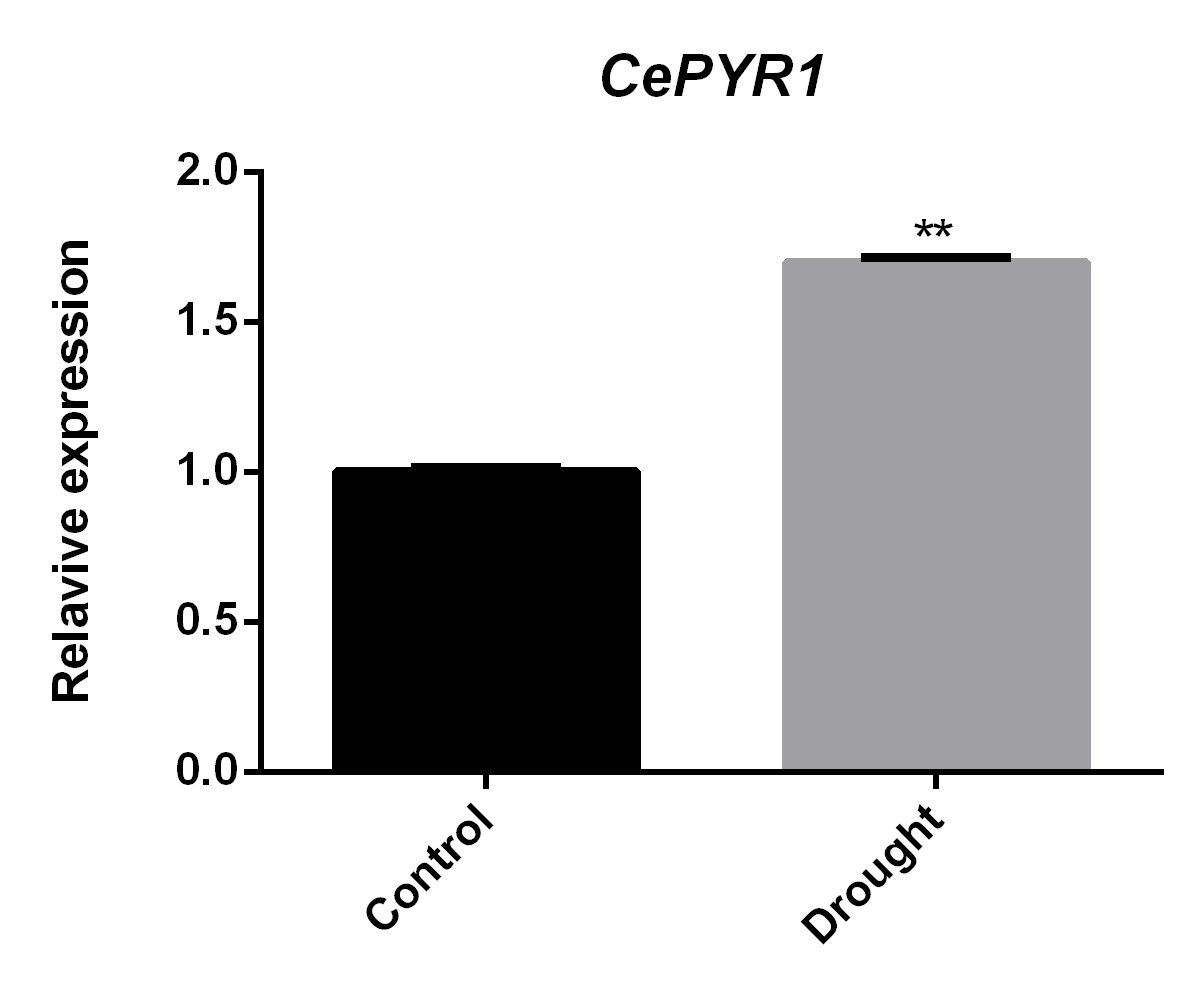


Figure S1. qRT-PCR analysis of *CePYR1* expression in leaves of tiger nut exposed to drought stress. Error bars represent the standard deviation. **significant difference at P < 0.01.
